# Role of miRNA-495 and NRXN-1 and CNTN-1 mRNA Expression and Its Prognostic Importance in Breast Cancer Patients

**DOI:** 10.1155/2021/9657071

**Published:** 2021-10-08

**Authors:** Ali G. Alkhathami, Amit Kumar Verma, Mohammed Alfaifi, Lalit Kumar, Mohammad Yahya Alshahrani, Abdulrahim R. Hakami, Osama M. Alshehri, Mohammed Asiri, Mirza Masroor Ali Beg

**Affiliations:** ^1^Department of Clinical Laboratory Sciences, College of Applied Medical Sciences, King Khalid University, Abha, Saudi Arabia; ^2^Department of Zoology and Environmental Sciences, GKV, Haridwar, India; ^3^Department of Cardiology, Sawai Man Singh Medical College, Jaipur, Rajasthan, India; ^4^Department of Clinical Laboratory Sciences, College of Applied Medical Science, Najran University, Najran, Saudi Arabia; ^5^Faculty of Medicine, Alatoo International University, Bishkek, Kyrgyzstan; ^6^Centre for Promotion of Medical Research, Alatoo International University, Bishkek, Kyrgyzstan

## Abstract

Breast cancer is a heterogeneous disease in which genetic factors are involved in disease worsening and higher mortality. Epidemiological and clinical research revealed that breast cancer incidence continues to rise. 100 histopathologically confirmed untreated newly diagnosed cases of invasive ductal carcinoma (IDC) of breast and 100 healthy subjects were involved and blood samples were collected in non-EDTA plain vials. Serum was separated by centrifugation, total RNA was extracted from serum, and cDNA synthesis was done to study the miRNA-495 and neurexin-1 (NRXN-1) and contactin 1 (CNTN-1) mRNA expression by QRT-PCR. The expression levels of miRNA-495, NRXN-1, and CNTN-1 were expressed in fold change. The present study observed decreased relative miRNA-495 expression (0.07-fold) while an increase in NRXN-1 (11.61-fold) and CNTN-1 (4.92-fold) was observed among breast cancer patients compared to healthy controls. A significant difference was observed in miRNA-495 expression with menopausal status (*p*=0.0001) and TNM stages (*p*=0.02). It was observed that NRXN-1 expression was significantly associated with menopausal status (*p*=0.03), lymph node involvement (*p* < 0.0001), estrogen receptor (ER) status (*p*=0.03), progesterone receptor (PR) status (*p*=0.005), TNM stages (*p* < 0.0001), and distant metastases (*p* < 0.0001). CNTN-1 expression was also found to be associated with lymph node involvement (*p*=0.01), PR status (*p*=0.03), HER2 status (*p*=0.04), TNM stages (*p* < 0.0001), and distant metastases (*p* < 0.0001). ROC suggested that NRXN-1 and CNTN-1 could be the important predictive marker for disease advancement and distant organ metastases. The study concluded that the decreased expression of miR-495 observed in breast cancer patients showed a negative correlation with NRXN-1 while the increased expression of NRXN-1 and CNTN-1 was linked with disease advancement and distant metastases and could be the important predictive marker for breast cancer patients.

## 1. Introduction

Breast cancer is one of the most common women's malignancies in the world and the treatment outcomes of patients remain poor. MicroRNAs (miRNAs) have emerged as promising therapeutic tools and targets, as they play important roles in regulating key cellular functions by interfering with gene expression [1]. miRNAs can control the gene expression programs in cells. It has been said that altered miRNAs expression significantly contributes to breast cancer development and progression [2] as the miRNAs are the crucial regulators of gene expression and play potential roles in the regulation of cancer cell progression [3]. BC expressing hormone receptor (estrogen receptor (ER+) or progesterone receptor (PR+)), BC expressing human epidermal receptor 2 (HER2+), and triple-negative breast cancer (TNBC) (ER−, PR−, HER2−) has been categorized based on histological evidence [4]. The treatment strategies should be based on the molecular characteristics of breast cancer and still the mechanisms of breast cancer origin is unknown [5]. As the world has moved toward targeted therapeutics for cancer patients, the proper management of early stage breast cancer patients has become increasingly important. [6, 7]. miRNA-495 is located on chromosome 14q32.31 [8] and it is involved in apoptosis, cell proliferation, and immune and inflammatory responses [9]. It has been discovered that abnormal miRNA-495 expression is linked to tumor cell proliferation, apoptosis, and chemoresistance [10]. Furthermore, as a tumor suppressor, miRNA-495 was found to be involved in the vast majority of solid tumors [11, 12]. miRNAs can regulate a large number of target genes, but they can be regulated by other multiple miRNAs in tumors depending on their regulatory functions [13] and miRNA-495 downregulation associated with cancer chemotherapeutic resistance [14]. The miRNA-495 is thought to play a role in the posttranscriptional regulation of NRXN-1 and CNTN-1 [15]. Several cell lines such as SHP77, NCI-H526, and HEK293 cells were used to analyze the neurexin-1 (NRXN-1) expression and it was found that the SHP77 had increased mRNA expression, while NCI-H526 showed moderate expression, and HEK293 showed decreased expression [16].

NRXN-1 expression was moderate in patient-derived cells (PDC), and surgical specimens revealed high NRXN-1 expression in a subset of primary SCLCs [16]. It is suggested that NRXN-1 is one of the key genes for glioma development and is linked to patient prognosis [17]. Contactin-1 (CNTN-1) is an immunoglobulin family member [18] and it has been demonstrated that CNTN-1 also plays a key role in esophageal squamous cell carcinoma (ESCC) [19], gastric carcinoma (GC) [20], lung adenocarcinoma [21], oral squamous cell carcinoma (OSCC) [22], hepatocellular carcinoma [23], and prostate cancer [24] progression and promotes the invasion and metastasis of cancer [25]. Therefore, the present study aimed to evaluate the prognostic importance of miRNA-495, NRXN-1, and CNTN-1 mRNA expression as well as the association of miRNA-495 expression with NRXN-1 and CNTN-1 mRNA expression among breast cancer patients.

## 2. Materials and Methods

### 2.1. Subject Recruitment, Blood Collection, and Total RNA Extraction

The present study included 100 histopathologically confirmed newly diagnosed untreated invasive ductal carcinoma (IDC) of breast cases and 100 healthy subjects. 4 ml of blood was collected in plain vials from all the study subjects. Further samples were centrifuged at 1500 rpm and the collected serum samples were stored at −80°C for further processing.

Total RNA extraction from the blood serum was performed using the TRIzol (Invitrogen) reagent following the manufacturer's instructions and stored at −80°C until an additional necessary step for cDNA synthesis. RNA quality and purity were determined using a nano spectrophotometer using the A260/280 ratio. The institutional ethical committee Gurukula Kangri University, Haridwar, India, approved this research study (Proposal no. 27/07/2017/GKV/IEC/2017) and informed consent was obtained from all the participants before the study was commenced at Zoology Department, Gurukula Kangri University.

#### 2.1.1. Polyadenylation and Complementary DNA Synthesis for miRNA-495

Following the manufacturer's protocol, 100 ng of total RNA was used for polyadenylation and cDNA synthesis using the TaqMan® Advance microRNA reverse transcription kit (TaqMan, Thermo Scientific). Universal RT primer and other essential reagents were added for cDNA synthesis to switch poly(*A*) tailed miRNAs into cDNA using a reverse transcriptase enzyme and other essential reagents provided with the manufacturer kit.

#### 2.1.2. QRT-PCR for miRNA-495 Expression

The serum-based miRNA-495 expression level was calculated using quantitative real-time PCR (qPCR). To calculate the expression, qPCR was performed with TaqMan master mix (4444556), TaqMan probes for miRNA-495 (478945 mir) for quantification, and U6(001973) as an internal control.

#### 2.1.3. Complementary DNA Synthesis and NRXN-1 and CNTN-1 mRNA Expression

100 ng of total RNA was used to synthesize the cDNA using the manufacturer-provided kit protocol (Verso, Thermo scientific, USA). NRXN-1 and CNTN-1 mRNA expression was evaluated by quantitative RT-PCR using SYBR Green I technology, and the GAPDH gene was used as the housekeeping control to analyze the fold change in mRNA expression.

Specific primer sequences were used to amplify NRXN-1 (forward: 5-CAGCAAAGCCTCTAACAGAAAAAGA-3; reverse: 5'-ACTGCTGCTTTGAATGGGGT-3') and CNTN-1 (forward: 5'-GTGGGAAACCTGTAGGGTATGG-3'; reverse 5'-TGTCTTCCTCAGAAACTCCATGA-3') and GAPDH (forward: 5'-GGTGGTCTCCTCTGACTTCAA -3'; reverse: 5'-GTTGCTGTAGCCAAATTCGTTGT-3'). The NRXN-1 and CNTN-1 mRNA expression studies were carried out using the program for 40 cycles, with the first denaturation step at 94°C for 40 s, annealing at various temperatures for 40 s, and extension at 72°C for 40 s, with the final reaction volume kept at 20 *μ*l and the final step in the extension process done at 72°C for 5 minutes. For target amplification, a melting curve analysis was performed between the temperatures of 35°C and 90°C, and all processes were duplicated to avoid errors. The NRXN-1 and CNTN-1 mRNA expression levels were calculated using the relative quantification method, 2^−(∆∆CT)^, with GAPDH as the in-house control, and the results were then expressed as the mean fold change in breast cancer patients compared to controls.

### 2.2. Statistical Analysis

Graph Pad Prism version 6.05 was used for all statistical analyses. The Mann–Whitney *U* test was used to determine whether there were any significant differences between the groups. The relative cycle threshold (Ct) method was used to analyze QRT-PCR data, with each sample being examined twice. The levels of miRNA-495, NRXN-1, and CNTN-1 expression were calculated using the 2^–(ΔΔCt)^ relative quantification method. Upregulation and downregulation of miRNA and mRNA expression, respectively, were determined by results greater than or less than 1. All of the results were standardized against the normal control values, which were given a value of 1, and *p* value < 0.05 was considered significant.

## 3. Results

### 3.1. General Characteristics of the Study Population

Demographic characteristics of 100 IDC of breast cases and 100 healthy controls are depicted in Table 1. Breast cancer patients and healthy controls were age-matched healthy controls. Among the breast cancer cases and healthy controls, 54% and 60% were in the age group of ≤50 years and 46% and 40% were in the age group of >50 years, respectively. In brief, other clinical features such as menopausal status, lymph node involvement, ER, PR, HER2, stage of disease, and metastatic condition of breast cancer cases are depicted in Table 1.

### 3.2. miRNA-495 Expression in Breast Cancer Cases and Comparison with Different Parameters

The present study observed 0.07-fold decreased relative miRNA-495 expression among breast cancer cases compared to healthy controls. It was observed that the cases with an age group of ≤50 years had 0.09-fold miRNA-495 expression while the age group of >50 years had 0.04-fold miRNA-495 expression (*p*=0.02). Breast cancer cases with menopausal status showed 0.04-fold miRNA-495 expression while cases with no menopausal status had 0.12-fold miR-495 expression and the differences among them were found to be statistically significant (*p*=0.0001). Advanced stage breast cancer cases showed 0.05-fold miRNA-495 expression while early-stage breast cancer cases showed 0.12-fold miRNA-495 expression (*p*=0.02) as depicted in Table 2.

### 3.3. NRXN-1 mRNA Expression in Breast Cancer Cases and Comparison with Different Parameters

Increased expression was observed among the breast cancer cases (11.61-fold) compared to healthy controls. Cases with the age group of ≤50 years had 8.75-fold NRXN-1 mRNA expression while the age group of >50 years had 14.98-fold NRXN-1 mRNA expression (*p*=0.0003). Breast cancer cases with positive menopausal status showed higher NRXN-1 mRNA expression (13.01-fold) compared to cases that do not have menopausal status (8.78-fold) and the expression differences among them was found to be statistically significant (*p*=0.03). Breast cancer patients with lymph node involvement had 15.70-fold higher NRXN-1 mRNA expression while breast cancer patients without any lymph nodes involvement had 8.40-fold NRXN-1 mRNA expression (*p* < 0.0001). Breast cancer patients who had an ER and PR positive status showed 14.05- (*p*=0.03) and 15.29-fold (*p*=0.005) NRXN-1 mRNA expression while ER and PR negative patients showed 10.52- and 10.18-fold NRXN-1 mRNA expression, respectively. Breast cancer cases in the advanced stage of disease had 16.78-fold NRXN-1 mRNA expression while early-stage breast cancer patients showed 3.18-fold NRXN-1 mRNA expression and differences between them were found to be statistically significant (*p* < 0.0001). It was found that the breast cancer distant metastatic patients showed 18.72-fold NRXN-1 mRNA expression while nonmetastatic patients showed 9.37-fold NRXN-1 mRNA expression (*p* < 0.0001) depicted in Table 3.

### 3.4. CNTN-1 mRNA Expression in Breast Cancer Cases and Comparison with Different Parameters

The relative gene expression of the CNTN-1 gene among the breast cancer cases was higher (4.92-fold) compared to healthy control. It was observed that the cases with the age group of ≤50 years had 4.35-fold CNTN-1 mRNA expression while the age group of >50 years had 5.60-fold CNTN-1 mRNA expression suggested to be lower comparatively (*p*=0.03). Breast cancer cases who had lymph node involvement had higher CNTN-1 mRNA expression (5.97-fold) compared to breast cancer cases who did not have lymph node involvement (4.10-fold) and the expression differences between them were found to be statistically significant (*p*=0.01). Breast cancer cases with positive PR status had 6.19-fold CNTN-1 mRNA expression while PR negative breast cancer cases had 4.44-fold CNTN-1 mRNA expression (*p*=0.03). HER2 positive breast cancer cases showed slightly higher expression of CNTN-1 mRNA expression (5.92-fold) while HER2 negative breast cancer cases had lower CNTN-1 mRNA expression (4.26-fold) comparatively (*p*=0.04). Advanced stage breast cancer cases had 6.89-fold CNTN-1 mRNA expression while being lower in the early-stage breast cancer cases (1.72-fold) and differences among them were found to be statistically significant (*p* < 0.0001). Cases with metastatic breast cancer had 8.85-fold CNTN-1 mRNA expression while nonmetastatic breast cancer cases had 3.68-fold CNTN-1 mRNA expression (*p* < 0.0001) as depicted in Table 4.

### 3.5. Correlation of miRNA-495 with NRXN-1 and CNTN-1 mRNA Expression among Breast Cancer Patients

**Figure 1:**
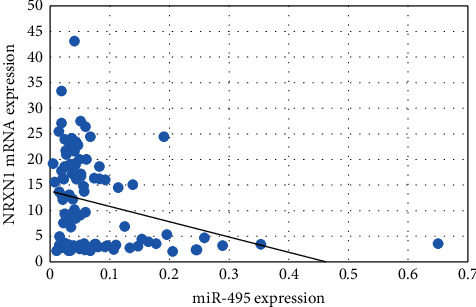
Correlation of miRNA-495 and NRXN-1 mRNA expression among the breast cancer cases.

**Figure 2:**
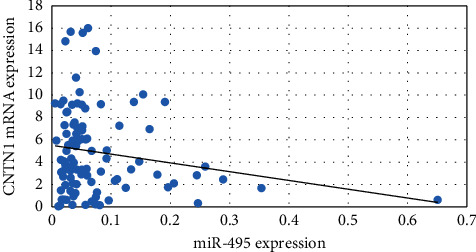
Correlation of miRNA-497 and CNTN-1 mRNA expression among the breast cancer cases.

Spearman correlation analysis was done between miRNA-495 expression with NRXN-1 (Figure 1) and CNTN-1 (Figure 2) mRNA expression among the breast cancer patients and it was observed that the miRNA-495 expression and NRXN-1 mRNA expression were negatively correlated (*r* = −0.23, *p*=0.01) while miRNA-495 expression and NRXN-1 mRNA expression did not show significant correlation (*r* = −0.08, *p*=0.41).

### 3.6. Correlation of NRXN-1 and CNTN-1 mRNA Expression among Breast Cancer Patients

**Figure 3:**
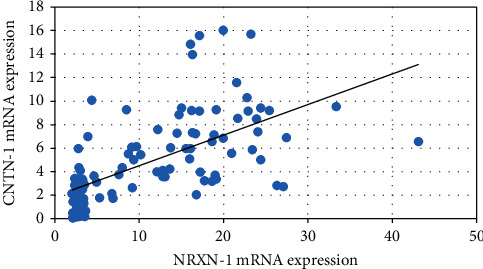
Correlation of NRXN-1 and CNTN-1 mRNA expression among the breast cancer cases.

Spearman correlation analysis was done between NRXN-1 and CNTN-1 mRNA expression (Figure 3) among breast cancer patients. There was a positive correlation observed (*r* = 0.61, *p* < 0.0001) suggesting with the increase of NRXN-1 mRNA expression; CNTN-1 expression was increased.

### 3.7. Prognostic Importance of miRNA-495, NRXN-1, and CNTN-1 mRNA Expression w.r.t. TNM Stages

**Figure 4:**
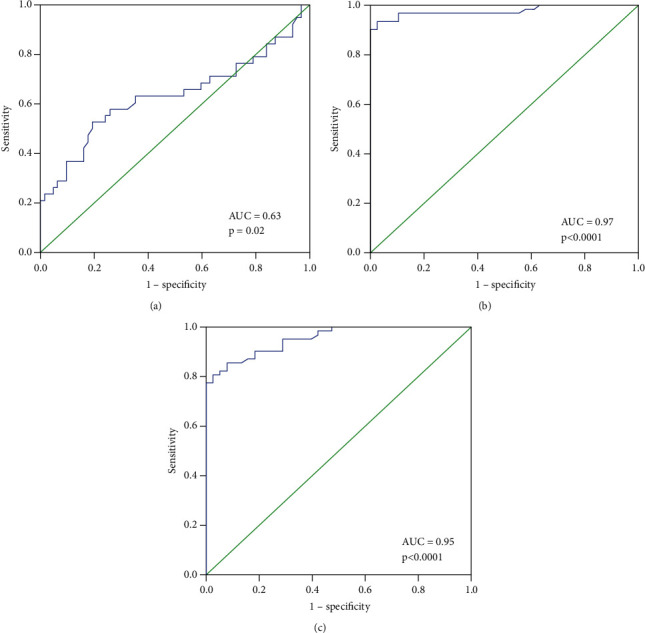
ROC curve: (a) microRNA-495, (b) NRXN-1, and (c) CNTN-1 w.r.t. TNM stages (early stage and advanced stage).

To examine the function of miRNA-495, NRXN-1, and CNTN-1 as predictive/prognostic biomarker for BC patients, TNM stages were categorized into two groups (early stage and advanced stage) and ROC curves analysis was performed (Table 5). The ROC curve was plotted between patients with early-stage breast cancer and those with advanced stage breast cancer. For ROC curves w.r.t early stage versus advanced stage of breast cancer at best possible cutoff value of the 0.03-fold decrease in miRNA-495 expression, the sensitivity and specificity were 65% and 62%, respectively (AUC = 0.63, *p*=0.02). For the ROC curve for the NRXN-1 w.r.t early stage versus advanced stage of breast cancer at the best possible cutoff value of a 4.5-fold increase in NRXN-1 expression, the sensitivity and specificity were 93% and 90%, respectively (AUC = 0.97, *p* < 0.0001) (Table 5) and for ROC curve for CNTN-1 w.r.t early stage versus advanced stage of breast cancer at best possible cutoff value of a 3.12-fold increase in CNTN 1 expression, the sensitivity and specificity were 90% and 82%, respectively (AUC = 0.95, *p* < 0.0001) (Figure 4).

### 3.8. Prognostic Importance of miRNA-495, NRXN-1, and CNTN-1 mRNA Expression w.r.t. Metastasis Status

To examine the function of miRNA-495, NRXN-1, and CNTN-1 as predictive/prognostic biomarker for BC patients, metastasis was divided into two groups, ROC curves were analyzed (Table 6), and BC patients with no metastases and those with distant organ metastases were plotted. ROC curves with respect to no metastases and distant organ metastases of breast cancer at the best possible cutoff value of a 0.04-fold decrease in miRNA-495 expression, sensitivity, and specificity were 57% and 46%, respectively (AUC = 0.57, *p*=0.27).

**Figure 5:**
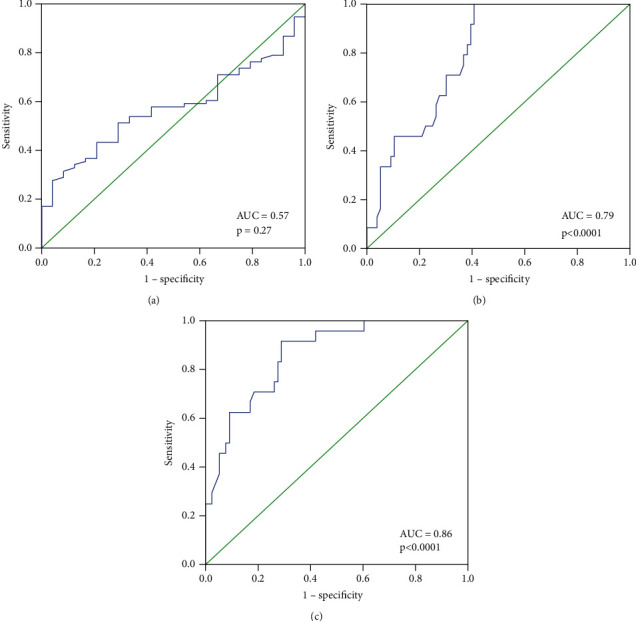
ROC curve: (a) microRNA-495, (b) NRXN-1, and (c) CNTN-1 w.r.t. metastases (no metastases and distant metastases).

For the ROC curve for no metastases and distant organ metastases of BC, the sensitivity and specificity of the NRXN-1 were 70% and 66%, at the best possible cutoff value of 13.36 -fold increase in NRXN-1 expression, respectively (AUC = 0.79, *p* < 0.0001) (Table 6) and for ROC curve for CNTN-1 w.r.t early stage versus advanced stage of breast cancer at the best possible cutoff value of a 5.7-fold increase in CNTN-1 expression, the sensitivity and specificity were 75% and 74%, respectively (AUC = 0.86, *p* < 0.0001) (Figure 5).

## 4. Discussion

In most countries, breast cancer (BC) is the leading cause of morbidity and mortality among women [26]. The rate of growth is rapidly increasing [27]. Early detection and treatment of BC (targeted therapies combined with chemoradiotherapy) have come a long way; however, more than half of cancer cells develop multidrug resistance quickly after chemoradiotherapy or multidrug resistant [28]. miRNA-495 has tumor suppressor activity in numerous cancer cells [29, 30] and Li et al. reported that hypermethylation could be the cause of expression suppression of miRNA-495 [31].

miRNA-495 levels were found to be significantly lower in breast cancer cell lines compared to NBECs, and ectopic expression of miRNA-495 significantly reduced proliferation and tumorigenicity in vitro and in vivo assays, suggesting that miRNA-495 downregulation may be linked to breast cancer characteristics [32]. It has also been mentioned that in gastric cancer, the expression level of miRNA-495 is downregulated [33] in prostate cancer [34], and non-small cell lung cancer [35]. However, it has been reported that miRNA-495 is directly involved in promoting cell invasion, which was further confirmed by Cao et al. who stated that miRNA-495 could induce breast cancer cell migration [36].

Formosa et al. reported downregulation of the miRNA-495 in metastatic prostate cancer cells [34]. Deregulation of miRNA-495 could influence the metastasis of NSCLC [37]. Additionally, in glioblastoma cells, loss of miRNA-495 promotes proliferation [38]. Finally, downregulation of miRNA-495 has been associated with tumorigenesis in metastatic prostate cancer [34]. Overall, these findings prompted us to look into the relationship between miRNA-495 and clinical characteristics like menopause, lymph node, ER, PR, HER2, TNM stages, and metastasis in large clinical samples of BC. The present study observed that breast cancer patients with menopausal status showed lower miRNA-495 expression compared to no menopausal status. It was also observed that the breast cancer patients in the advanced stage (0.05-fold) and distant organ metastases (0.04-fold) had lower miRNA-495 expression compared to the early stage (0.12-fold) and no distant organ metastases (0.08-fold) breast cancer patients. Correlation analysis showed a significant negative correlation between miRNA-495 expression and NRXN-1 mRNA expression among the breast cancer patients while a positive correlation between NRXN-1 and CNTN-1 mRNA expression was observed.

NRXN-1 mRNA expression was analyzed in small cell lung cancer cell line such as SHP77 and NCI-H526 and it was found that the SHP77 had a higher mRNA expression, while NCI-H526 had comparatively low mRNA expression [16]. It was revealed that PDC showed a reasonable NRXN-1 expression level while surgical specimens subset of primary SCLCs had higher NRXN-1 mRNA expression [16].

Yotsumoto et al. suggested that knockout of NRXN-1 in SHP77 cells resulted in a loss of the antitumor activity of NRXN-1 [16]. It has been suggested that NRXN-1 is the key gene for glioma development and is related to the prognosis of patients [17]. The present study observed higher mRNA expression of NRXN-1 in breast cancer patients with higher age group (>50 years) menopausal status, patients with lymph node involvement compared to its counterpart. Higher NRXN-1 mRNA expression was observed to be linked with the positive ER and PR status among breast cancer patients. It was also found that the higher expression of NRXN-1 was associated with the advancement of the disease and distant organ metastases among breast cancer patients. ROC showed that NRXN-1 had good sensitivity and specificity at the cutoff value of 4.5-fold NRXN-1 mRNA expression and could be used as a prognostic marker for the advancement of the disease and at the cutoff value of 13.36-fold NRXN-1 mRNA expression for the prediction of distant organ metastases.

Abnormal CNTN-1 expression has been linked to pathological phenotypes like cell proliferation, invasion, metastasis, and poor prognosis [39]. Knockdown of CNTN-1 reduced cell invasion but not proliferation [40]. Through activation of the phosphatidylinositol 3-kinase (PI3K)/AKT signaling pathway, CNTN-1 promotes cisplatin resistance in cisplatin-resistant lung cancer cells and resistant cells. Knocking down CNTN-1 partially reversed the epithelial-mesenchymal transition (EMT), improved drug sensitivity, and slowed the progression of cancer [41]. CNTN-1 was upregulated in BCT-100-resistant cell lines and promoted EMT progression via the AKT pathway. The CNTN-1 gene was silenced, which resensitized resistant cells to BCT-100 treatment and reduced the EMT phenotype [42]. CNTN-1 mRNA was found to be upregulated in gastric tumors compared to noncancerous gastric samples, and this upregulation was linked to tumor size, TNM stages, metastases, and invasion [43]. HCC had higher CNTN-1 mRNA and protein expression than adjacent tissues, which was linked to tumor size and metastasis [44]. Overexpression of CNTN-1 promoted tumor growth by enhancing breast cancer cell proliferation, migration, invasion, and cell cycle progression [45]. In the same way, our findings suggested that higher expression of CNTN-1 is linked with lymph node involvement, PR status, and PR positive status. It was also found that higher CNTN-1 mRNA expression is linked with the advancement of the disease and distant organ metastases. ROC curve analysis also showed that the CNTN-1 at a cutoff value of 3.12-fold had good sensitivity and specificity and could be used as a predictive/prognostic marker for the advancement of disease, and at a cutoff value of 5.7-fold, CNTN-1 expression also can be used for the prediction of distant organ metastases.

## 5. Conclusion

This study concludes that the miRNA-45 was downregulated showing a negative correlation with NXRN-1 among breast cancer patients. Higher expression of NXRN-1 and CNTN-1 mRNA was linked with disease advancement and distant metastases. ROC curve suggested that NXRN-1 and CNTN-1 could be used as a prognostic/predictive marker for disease advancement and distant metastases.

## Figures and Tables

**Table 1:** Demographic and clinical characteristic of breast cancer cases.

Variables	Breast cancer cases 100 (%)	Healthy controls 100 (%)
*Age*		
≤50 years	54 (54)	60 (60)
>50 years	46 (46)	40 (40)

*Menopause*		
Yes	67 (67)	60 (60)
No	33 (33)	30 (30)

*Lymph nodes*		
Yes	44 (44)	
No	56 (56)	

*ER status*		
Yes	31 (31)	
No	69 (69)	

*PR status*		
Yes	28 (28)	
No	72 (72)	

*HER2 status*		
Yes	40 (40)	
No	60 (60)	

*TNM stages*		
Early stage (I & II)	38 (38)	
Advanced stage (III & IV)	62 (62)	

*Distant metastases*		
Yes	24 (24)	
No	76 (76)	

**Table 2:** Association of miRNA-495 expression in breast cancer cases with different clinicopathological features.

Variables	miRNA-495 expression		*p* value
	Mean	SD	
Relative expression	0.07	0.05	—

*Age*			
≤50 years	0.09	0.11	0.02
>50 years	0.04	0.02	

*Menopause*			
Yes	0.04	0.03	0.0001
No	0.12	0.12	

*Lymph nodes*			
Yes	0.07	0.10	0.21
No	0.07	0.07	

*ER status*			
Yes	0.05	0.04	0.10
No	0.09	0.10	

*PR status*			
Yes	0.05	0.05	0.11
No	0.08	0.09	

*HER2 status*			
Yes	0.05	0.04	0.49
No	0.08	0.10	

*TNM stages*			
Early stage (I & II)	0.12	0.11	0.02
Advanced stage (III & IV)	0.05	0.03	

*Distant metastases*			
Yes	0.04	0.02	0.27
No	0.08	0.09	

**Table 3:** Association of NRXN-1 mRNA expression in breast cancer cases with different clinicopathological features.

Variables	NRXN-1 mRNA expression		*p* value
	Mean	SD	
Relative expression	11.61	7.64	—

*Age*			
≤50 years	8.75	7.98	0.0003
>50 years	14.98	8.71	

*Menopause*			
Yes	13.01	8.86	0.03
No	8.78	8.23	

*Lymph nodes involvement*			
Yes	15.70	8.96	<0.0001
No	8.40	7.37	

*ER status*			
Positive	14.05	7.83	0.03
Negative	10.52	9.11	

*PR status*			
Positive	15.29	7.43	0.005
Negative	10.18	8.98	

*HER2 status*			
Positive	12.71	8.36	0.20
Negative	10.88	9.15	

*TNM stages*			
Early stage (I & II)	3.18	1.04	<0.0001
Advanced stage (III & IV)	16.78	7.41	

*Distant metastases*			
Yes	18.72	8.28	<0.0001
No	9.37	7.81	

**Table 4:** Association of CNTN-1 mRNA expression in breast cancer cases with different clinicopathological features.

Variables	CNTN-1 mRNA expression		*p* value
	Mean	SD	
Relative expression	4.92	3.02	—

*Age*			
≤50 years	4.35	3.88	0.03
>50 years	5.60	3.55	

*Menopause*			
Yes	4.98	3.68	0.72
No	4.81	3.99	

*Lymph nodes*			
Yes	5.97	3.99	0.01
No	4.10	3.39	

*ER status*			
Yes	5.97	4.0	0.06
No	4.45	4.59	

*PR status*			
Yes	6.19	3.90	0.03
No	4.44	3.62	

*HER2 status*			
Yes	5.92	4.21	0.04
No	4.26	3.30	

*TNM stages*			
Early stage (I & II)	1.72	1.24	<0.0001
Advanced stage (III & IV)	6.89	3.43	

*Distant metastases*			
Yes	8.85	3.92	<0.0001
No	3.68	2.74	

**Table 5:** ROC curve for microRNA-495, NRXN-1, and CNTN-1 w.r.t. TNM stages (early stage and advanced stage).

AUC for microRNA-495 (95% CI)	Sensitivity	Specificity	Cutoff value (fold change)	*p* value
0.63 (0.50–0.75)	65%	62%	0.03-fold	0.02

AUC for NRXN-1 (95% CI)	Sensitivity	Specificity	Cutoff value (fold change)	*p* value
0.97 (0.94–1.0)	93%	90%	4.5-fold	<0.0001

AUC for CNTN-1 (95% CI)	Sensitivity	Specificity	Cutoff value (fold change)	*p* value
0.95 (0.91–0.98)	90%	82%	3.12-fold	<0.0001

**Table 6:** ROC curve for microRNA-495, NRXN-1, and CNTN-1 w.r.t. metastases (no metastases and distant organ metastases).

AUC for microRNA-21 (95% CI)	Sensitivity	Specificity	Cutoff value (fold change)	*p* value
0.57 (0.46–0.68)	57%	46%	0.04-fold	0.27

AUC for NRXN-1 (95% CI)	Sensitivity	Specificity	Cutoff value (fold change)	*p* value
0.79 (0.70–0.88)	70%	66%	13.36-fold	<0.0001

AUC for CNTN-1 (95% CI)	Sensitivity	Specificity	Cutoff value (fold change)	*p* value
0.86 (0.78–0.93)	75%	74%	5.7-fold	<0.0001

## Data Availability

The datasets used and/or analyzed during the present study can be available from the corresponding author if needed.
